# Special sites in atopic dermatitis: Effectiveness of dupilumab on the hands in a single‐centre study on 485 patients

**DOI:** 10.1111/ajd.14372

**Published:** 2024-10-21

**Authors:** Gabriele Perego, Italo Francesco Aromolo, Francesca Barei, Martina Zussino, Luca Valtellini, Angelo Valerio Marzano, Silvia Mariel Ferrucci

**Affiliations:** ^1^ Department of Pathophysiology and Transplantation University of Milan Milan Italy; ^2^ Dermatology Unit Foundation IRCCS Ca’ Granda Ospedale Maggiore Policlinico Milan Italy

**Keywords:** atopic dermatitis, dermatological therapy, dupilumab, hands, quality of life

Approximately 60% of atopic dermatitis (AD) involve the hands.^1^ As exposed areas, the hands are susceptible to physical and chemical factors, such as low temperatures, allergens, UV rays, and irritants. These factors can damage the skin barrier, promoting the inflammatory flare‐ups of AD and making this area potentially more resistant to treatment.^2^ Hands are essential tools for daily activities, and the presence of AD in this area significantly impacts the patient's quality of life (QoL).^3^ Dupilumab, a monoclonal antibody targeting IL‐4 and IL‐13 signalling, is highly effective for treating AD, although its specific efficacy on the hands has been minimally assessed in the literature.^4^, ^5^, ^6^, ^7^, ^8^, ^9^

A single‐centre, retrospective, study was conducted on 485 patients with severe AD involving the hands, all of whom were treated with dupilumab (loading dose of 600 mg, followed by 300 mg every 2 weeks via subcutaneous injections). Two hundred and fifty‐five were male (52.5%), with a mean age of 38 years at the start of treatment (min–max, 13–88). In patients with suspected allergic contact dermatitis superimposed on AD, patch tests were performed, and if positive, the patients were excluded from the study. For clinical assessment, the Eczema Area and Severity Index (EASI), Pruritus Numerical Rating Scale (NRS), Atopic Dermatitis Control Tool (ADCT) and Dermatology Life Quality Index (DLQI) were used. Data were collected at baseline, and every 4 months during treatment. Complete remission (CR) was defined as an EASI score = 0 and Pruritus NRS = 0 at the follow‐up visit in a patient not using topical corticosteroids or calcineurin inhibitors in the previous 4 months. Patients enrolled in the study were allowed to use emollient creams throughout the observation period. A *t*‐test or Mann–Whitney *U*‐test was used, as appropriate, to investigate potential differences in ADCT and DLQI scores between clinical groups. All statistical analyses were two‐tailed, with an alpha error = 0.05. A *p* < 0.05 was considered significant.

After 4 months of therapy, 62.7% of patients achieved CR in the hands. This response rate increased to 76.5% after 1 year and 85.6% after 3 years of treatment (Figure 1). In all comparisons (M4, M12 and M24), the DLQI and ADCT scores were significantly higher in non‐responder patients compared to responders, except for the comparison of DLQI scores at 4 months (Table 1).

**FIGURE 1:**
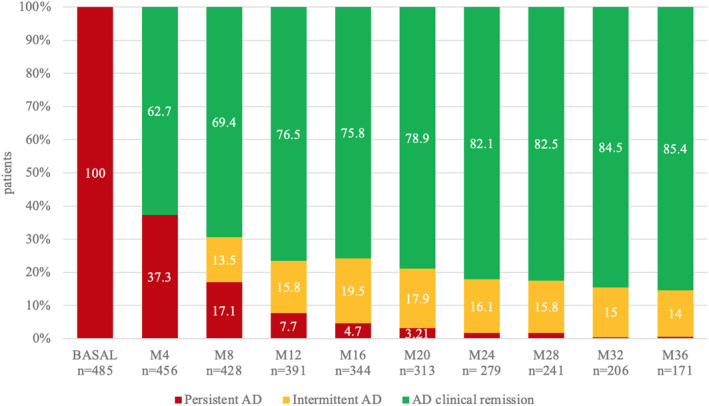
During treatment with dupilumab in AD, hands involvement at different follow‐up times, classified as persistent AD, intermittent AD and complete remission in that area. Patients were classified as follows: Persistent AD (if the hands remained consistently affected), intermittent AD (if the area alternated between periods of involvement and remission in the previous follow‐up visits) and clinical remission (if the area experienced complete persistent complete remission after the initial clearance). Not all patients were followed up for 3 years, as they were enrolled at different times. AD, atopic dermatitis; M*X*, follow‐up after *X* months (e.g. M4, at 4 months of follow‐up); n, number of patients evaluated at each follow‐up. Data not written in Figure 1: Patients with persistent AD are 1.8% at M24, 1.7% at M28, 0.5% at M32 and 0.6% at M36.

**TABLE 1:** DLQI and ADCT scores in patients with hands involvement at 4, 12 and 24 months of treatment with dupilumab.

	Responder	Non‐responder	*p*‐Value
M4			
DLQI, mean (SD)	4.65 (±4.85)	5.21 (±4.88)	0.208
ADCT, mean (SD)	5.61 (±4.15)	6.60 (±3.87)	0.007
M12			
DLQI, mean (SD)	3.27 (±4.09)	4.25 (±4.12)	0.0035
ADCT, mean (SD)	3.99 (±3.88)	5.79 (±4.22)	0.0001
M24			
DLQI, mean (SD)	2.38 (±2.88)	3.80 (±3.38)	0.002
ADCT, mean (SD)	3.65 (±3.66)	4.94 (±3.74)	0.021

*Note*: We considered patients with a complete persistent clinical remission as responders and patients with any degree of hands involvement in atopic dermatitis as non‐responders.

Abbreviations: ADCT, Atopic Dermatitis Control Tool; DLQI, Dermatology Life Quality Index; mean (±SD), mean value and standard deviation; MX, Follow‐up after X months; NR‐MX, non‐responder patients at follow‐up after X months; R‐MX, responder patients at follow‐up after X months.

Phase III clinical trials have demonstrated the effectiveness of dupilumab in treating AD across different anatomical regions; however, the hands have not been specifically considered.^10^ The evidence is based on real‐life data: Vittrup et al. observed that 65% of 104 patients with AD achieved CR after 1 year of treatment with dupilumab—a percentage similar to or even better than that observed in other areas.^4^ Other smaller case studies confirm these findings.^5^, ^6^, ^7^, ^8^ A recently published placebo‐controlled trial demonstrated that dupilumab led to clinically meaningful improvements in 67 patients with hand and foot AD^9^. However, in that study, some patients with chronic hand eczema were also included, and the treatment duration was relatively short (16 weeks).

Our study confirms the effectiveness of dupilumab for the hands in a large sample, with 85% of 485 patients achieving CR after a prolonged treatment period (3 years). Among patients who did not reach CR, the percentage with persistent dermatitis decreased over time in favour of an intermittent form (Figure 1). Prolonging treatment may benefit a subgroup of initially unresponsive patients, both in achieving CR and in transitioning from a persistent into an intermittent form. This could suggest not discontinuing dupilumab prematurely in favour of alternative therapeutic agents.

The effectiveness of dupilumab is particularly relevant in the treatment of chronic hand eczema, a distinct but partially overlapping entity with AD involving the hands. The promising results in treating chronic hand eczema with dupilumab^9^, ^11^ further supports the idea of hands as a site prone to therapeutic responses, despite their traditional identification as a difficult‐to‐treat site.

The presence of AD in the hands is associated with a lower QoL,^3^ due to its impact on daily activities and social relationships. The placebo‐controlled trial by Simpson et al.^9^ showed that patients with hands AD receiving dupilumab experienced a significant improvement from baseline in the QoL Hand Eczema Questionnaire (QoLHEQ), a site‐specific questionnaire. Our study demonstrated that DLQI and ADCT scores remained consistently higher in non‐responders, underscoring the importance of hands in influencing outcomes even in more general QoL measures.

## AUTHOR CONTRIBUTIONS

Italo Francesco Aromolo and Gabriele Perego equally participated in data acquisition, analysis, interpretation and drafting of the manuscript. Francesca Barei and Luca Valtellini participated in drafting the manuscript. Silvia Mariel Ferrucci, Martina Zussino and Angelo Valerio Marzano participated in study concept and design and supervised the study. All authors critically revised the manuscript for important intellectual content and approved the final manuscript.

## CONFLICT OF INTEREST STATEMENT

SM Ferrucci is principal investigator in clinical trial to Amgen, Sanofi, Novartis, Lilly, Leo Pharma, Abbvie and she is advisory board or speaker to Novartis, Menarini, Sanofi, Abbvie and Leo Pharma. The other authors declare that there is no conflict of interest.

## FUNDING INFORMATION

None.

## ETHICS STATEMENT

The study was conducted in accordance with the ethical standards of the responsible committee on human experimentation (institutional and national), with the Helsinki Declaration of 1975, as revised in 2000, and with the Taipei Declaration.

## PATIENT CONSENT STATEMENT

Written informed consent was obtained from the patient included in the study.

## Data Availability

Anonymized data will be shared upon reasonable request from any qualified investigator for purposes of replicating procedures and results.
